# Simulation Modelling of Population Dynamics of Mosquito Vectors for Rift Valley Fever Virus in a Disease Epidemic Setting

**DOI:** 10.1371/journal.pone.0108430

**Published:** 2014-09-26

**Authors:** Clement N. Mweya, Niels Holst, Leonard E. G. Mboera, Sharadhuli I. Kimera

**Affiliations:** 1 National Institute for Medical Research, Tukuyu, Tanzania; 2 Department of Agroecology, Aarhus University, Slagelse, Denmark; 3 National Institute for Medical Research, Dar es salaam, Tanzania; 4 Department of Veterinary Medicine and Public Health, Sokoine University of Agriculture, Morogoro, Tanzania; Division of Clinical Research, United States of America

## Abstract

**Background:**

Rift Valley Fever (RVF) is weather dependent arboviral infection of livestock and humans. Population dynamics of mosquito vectors is associated with disease epidemics. In our study, we use daily temperature and rainfall as model inputs to simulate dynamics of mosquito vectors population in relation to disease epidemics.

**Methods/Findings:**

Time-varying distributed delays (TVDD) and multi-way functional response equations were implemented to simulate mosquito vectors and hosts developmental stages and to establish interactions between stages and phases of mosquito vectors in relation to vertebrate hosts for infection introduction in compartmental phases. An open-source modelling platforms, Universal Simulator and Qt integrated development environment were used to develop models in C++ programming language. Developed models include source codes for mosquito fecundity, host fecundity, water level, mosquito infection, host infection, interactions, and egg time. Extensible Markup Language (XML) files were used as recipes to integrate source codes in Qt creator with Universal Simulator plug-in. We observed that Floodwater Aedines and Culicine population continued to fluctuate with temperature and water level over simulation period while controlled by availability of host for blood feeding. Infection in the system was introduced by floodwater Aedines. Culicines pick infection from infected host once to amplify disease epidemic. Simulated mosquito population show sudden unusual increase between December 1997 and January 1998 a similar period when RVF outbreak occurred in Ngorongoro district.

**Conclusion/Significance:**

Findings presented here provide new opportunities for weather-driven RVF epidemic simulation modelling. This is an ideal approach for understanding disease transmission dynamics towards epidemics prediction, prevention and control. This approach can be used as an alternative source for generation of calibrated RVF epidemics data in different settings.

## Introduction

Rift Valley fever (RVF) is an infection caused by arbovirus belonging to genus *Phlebovirus* of the family *Bunyaviridae*. The viruses use arthropod vectors such as mosquitoes and sand flies for infection transfer to livestock and humans [1]. Since its first description in 1930 in Kenya [2], [3], the virus has occurred as epidemic disease in Sub-Saharan Africa primarily in eastern and southern Africa, North Africa, Arabian Peninsula and Madagascar [4], [5] and poses a potential threat to Europe [6]. In all recorded epidemics, the disease had socio-economic impact due to high animal and human morbidity and mortality. The major outbreaks in Kenya, Tanzania and Somalia were in 1997–1998 and 2006–2007 [7], [8], with human deaths totalling 478 and 318 in years 1998 and 2007 respectively [9]. During the 2006–2007 outbreaks in Kenya and Tanzania, a reported number of 16,973 cattle, 20,193 goats, and 12,124 sheep died of the disease, with spontaneous abortions observed for 15,726 cattle, 19,199 goats, and 11,085 sheep [9]–[11]. Similar to other arboviral infection, RVF virus is passed from generation to generation of Aedine mosquitoes trans-ovarially [12]–[14]. This vertical disease transmission permits the virus to survive over prolonged periods because eggs can survive for several years in dry conditions [12], [15]–[17].

Emergence of infected mosquito populations and amplification of the virus are determined by changes in weather conditions [15], [18], [19]. In East Africa, RVFV epidemics are known to be associated with patterns of unusually heavy rainfall [20]. This led the World Health Organization (WHO) and Food and Agriculture Organization (FAO) to developed RVF forecasting models centred on cyclical patterns of the El Niño/Southern Oscillation (ENSO) [18], [21]. These models incorporate measurements of global and regional elevated sea surface temperatures, rainfall and satellite derived-normalized difference vegetation index data [22]–[24] which derive from Remote Sensing Satellite Imagery (RSSD), including use of Landsat, SPOT and Synthetic Aperture Radar and Cold Cloud Density (CCD) which allow use of more sophisticated tools to predict RVF virus epizootic activity over much wider areas [24]–[26]. Predictions were corroborated through entomological field investigations of mosquitoes and virus activity in the suspected area [27] as a key element in controlling RVF [28]. However, recent climate-driven prediction results in 2012 for some areas in Kenya and Tanzania [29] indicating foreseeable challenges due to the complexity of the disease (virus, vectors, and hosts) involved and their interactions with the environment hence a need to incorporate more tools.

Mathematical models have been developed for RVFV epidemics to complement available weather only dependent prediction models [30]–[33]. Many of them are based on previously developed epidemiological model of RVF that focus mainly on animals and vectors population dynamics with hypothetical consideration of infection dynamics [32]. Further development of this model incorporated the role of vaccination and vector control to describe epidemiology of RVFV in areas of intense transmission [34], [35]. Other developments for this model associate exclusion of a vertical transmission in vectors and inclusion of animal movements for spatial spread of disease [36]–[38]. Some models associate epidemics with cryptic cycles of the virus within animal hosts [39] and a more improved vertical transmission in vectors that include seasonality [31]. The role of daily weather data such as temperature and rainfall as model input to determine vector populations have not previously been directly considered. This limits their further applicability in predictive epidemiology due to insufficient incorporation of weather data and on-the-ground biological processes related to RVF disease.

Development of prediction models for RVFV epidemics faces many challenges like lack of reliable data. Absence of field-based rapid diagnostic tools results in the disease first being detected when it is actually beginning to decline from within the infected populations. RVF epidemics preparedness teams are therefore less effective for counter-measure against the impact of the disease. It is well documented that in order for disease to be controlled by vaccination, animals need to be vaccinated 4–6 weeks before stress and risk periods [40] to ensure that the vaccinated population have developed enough immunity against the virus [29], [41]. We therefore present a simulation modelling approach that incorporate weather data to simulate on-the-ground entomological data on mosquito abundances in relation to their hosts as previously recommended [11].

## Materials and Methods

### Study scenario and data source

The Ngorongoro district in Tanzania was purposely selected as the main study scenario. The district is part of the Serengeti-Masai Mara Ecosystem, which is defined by the limits of the annual wildlife migration linking with a neighbour country, Kenya experiencing similar disease epidemics. The district represents unique interaction between livestock, wildlife and human interface with animal migrations. The area has experienced several records of RVFV outbreaks. According to 2006–2007 outbreaks, high animal mortality was recorded in this area [5]. Freely accessible daily rainfall and temperature data from 1994–1999 for Ngorongoro with Narok ecosystem, Mwanza and Musoma regions downloaded from http://www.ncdc.noaa.gov/cdo-web.

### Assumptions for simulation model

**Figure 1:**
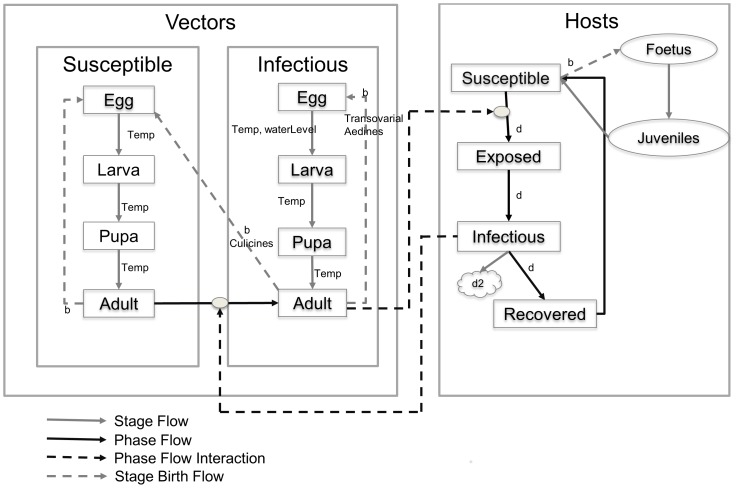
Diagrammatic presentation of RVF vector population dynamics simulation model. Adult mosquitoes lay eggs directly in breeding sites or in soil above water level (the latter remain inactive for many years). Hatching of inactive floodwater Aedes eggs depend on water level in breeding sites which in-turn depends on amount of daily rainfall. Our model considers mosquito growth and mortality in each developmental stage depend on temperature, water level and host availability. Mosquitoes move from susceptible to infectious phase after contact with infectious host. Hosts remain in the susceptible phase until after effective contact with infectious mosquitoes, and then hosts flow from susceptible to exposed, infectious and recovered phases. **Abbreviations**: b = births, d = natural mortality, d_2_ = mortality due to disease, temp = depends on temperature, waterLevel = depends on water in breeding sites, transovarial = transovarial transmission.

The assumptions for model development include; Floodwater Aedine mosquitoes are responsible for maintenance of the virus with vertical transmission and Culicine mosquitoes play a major role in virus amplification during epidemics. Water level in potential breeding sites determines hatching of Floodwater Aedine eggs. Increased water level in breeding sites is required to allow infected eggs laid further in the soil to hatch. Mosquito developmental stages use cumulative temperature as important model input factor to determine maturation time and adults survival. Mosquito search for a blood meal is a function of availability of hosts and that the probability of a successful blood meal is a function of the availability of host. During feeding, a mosquito has the probability of transferring viral infection to a host, or becoming infected by taking a blood meal from a viremic host. Flow of infection in mosquitoes and host is governed in compartmental phases. RVFV infection is initiated in a single phase small population of Floodwater Aedine mosquitoes before reaching the amplifying Culicine mosquitoes (Figure 1).

### Formulation for simulation model

1-D and 2-D time-varying distributed delay (TVDD) equations were used to formulate the models [42]–[45]. These time-variant distribution delay equations were initially developed in 1970s based on a kth order time-invariant distributed delay and later on applied as stage structured population dynamics models. TVDD models emphasize that delay in the distribution from one stage or phase to another is the quality of the output given some input parameters. All entities that enter the delay process at the input either leave at the output or remain stored inside the process. The 2-D TVDD are implemented similar to 1-D TVDD in a way that stages and phases are capable of interacting simultaneously [46]. Details on how these mathematical models were implemented in C++ programming language are indicated in Text S1. Four developmental stages such as eggs, larvae, pupae and adults with host age groups were modelled using 1-D TVDD. 2-D TVDD were used to model distribution compartmental phases of mosquitoes and hosts. Mosquitoes were categorised in two phases; Susceptible (S) and Infectious (I). Hosts were categorised in four phases; Susceptible (S), Exposed (E), Infectious (I) and Recovered (R) (Figure 1). We have included the exposed stage because we need model predictions to be accurate to the nearest day by accounting for the time lag between infection and the onset of infectiousness.

During each mosquito developmental stage temperature dependence delays were used [44], [45]. The mean delay time for mosquitoes to pass through a stage of growth is calculated as a total required number of degree days given as cumulative temperature. A simplified water balance model was used to simulate daily variations in amount of water in potential breeding sites for floodwater Aedine mosquitoes. Daily rainfall data was used as model input to determine cumulative amount of water in breeding sites after deduction of daily water loss due to other factors such as evaporation. We applied water balance equation that uses the principles of conservation of mass in a closed system as previously described [47], [48] but with added simplicity to reflect mosquito breeding behaviour. Cumulative amount of water in breeding sites determined hatching of floodwater Aedine eggs laid on the soil above water level in breeding sites (Figure 1).

Multi-way functional response equations previously described for predator prey relations [49] were modified to reflect vector-host interactions in a disease setting as indicated in Text S1. Mult-way functional responses were used to determine how host search for blood meal influenced mosquito fecundity. Mosquito vector phases (susceptible or infectious) were allowed to take a blood meal from all phases and stages of a host. Interaction between infectious mosquito vectors with susceptible host caused a phase outflow to exposed hosts. Interaction between susceptible mosquito vectors with infectious host caused a phase outflow to infectious vectors. Infectious Floodwater Aedine mosquitoes were allowed to lay infectious eggs timed to hatch depending on cumulative water level in breeding sites above threshold. Infectious Culicines laid eggs hatching susceptible mosquitoes as they lack transovarial transmission. Infection transfer to hosts was calculated automatically as indicated in Text S1. Phase outflow for the host from exposure phase to infectious and then recovered take consideration of host mortality due to disease and recovered host were allowed to flow into a susceptible phase (Figure 1). RVFV don’t induce lifelong immunity like measles, recovered animals should be at risk of getting infection again but it is still not known how long it takes before they become susceptible again.

### Development of simulation model

Model algorithms were developed using an open-source Universal Simulator and Qt Integrated Development Environment in C++ programming language [50]. We followed procedures for installation and use of Qt creator, Universal Simulator end user and developer’s versions as provided in the Universal Simulator website (http://www.ecolmod.org). Source codes for mosquito fecundity, host fecundity, water level, mosquito infection, host infection and Aedines eggs time were prepared. Extensible Markup Language (XML) files were prepared as recipe to integrate source codes in Qt creator with Universal Simulator plug-in. Source codes incorporated control structures for hatching of infectious inactive egg laid in the soil due to water level increase in breeding sites and RVFV infection initiation from Floodwater Aedine mosquitoes to susceptible host and then to Culicine mosquitoes for virus amplification. Details of parameters used are as shown in Table 1 and source codes are shown in Text S1.

**Table 1:** Parameters description.

Parameter	Description	Value range	Details	Reference
aedesLongevity	Longevity of females Aedes	30.0–45.8 days		[58]
aedesFecundity	Eggs laid per female Aedes per day	25–35 egg per day		[59]
aedesEggMortality	Number eggs dead per day in a stage	11.3–12.9%	failure to hatch	[55], [59], [60]
aedesHatchRate	Hatch rate for Aedes	85–95%		[59]
aedesLarvalSurvival	Larvae survival rates	90–100%		[59]
aedesLarvaeMortality		13.97–16.7%	Endogenous causes	[59]
aedesPupaMortality		17–30%	Endogenous causes	[59]
sexRatio	Proportion of female mosquitoes for both Aedes and Culex spp	1∶1		[58], [59]
transovarial	Virus transovarial transmission rate	Range from 0 to 1	RVF virus vertical transmission in Aedes mosquitoes is still not known	[31]
gonotrophicCycles	Gonotrophic cycles or number of blood meals per female	5–9 times	Cycle after every 3–5 days	[58]
culexLongevity	Survival or longevity (females Culex)	25.2–36.9 days		[61], [62]
culexFecundity	Culex fecundity	21–69 eggs per day	63–200 eggs after every 3 to 5 days	[63]
culexHatchRate	Egg hatching (Culex)	75.2–89.0%		[62]
culexEggMortality	Egg mortality (Culex)	10.9–24.9%		[62]
culexPupaGrowth	Larva pupation (Culex)	47.8–68.5%		[62]
culexLarvaeMortality	Larvae mortality (Culex)	15.9–31.8%		[62]
culexPupaMortality	Pupae mortality (Culex)	5.62–6.13%		[62]
activationRate	Infectious eggs hatch from soil per day	10–100%		[64]
waterLevelThreshold	Minimum amount of water in mosquito breeding sites required to activate infectious eggs		Adjusted based on parameter sensitivity analysis	
dailyLoss	Amount of water lost per day	Fixed/Manually adjusted	Adjusted based on parameter sensitivity analysis	
daysDegreesLarvae	Cumulative temperature for larvae growth	206 Celsius degrees		[55], [59], [60], [65]–[67]
daysDegreesPupae	Cumulative temperature for pupa growth	74 Celsius degrees		[55], [59], [60], [65]–[67]
sheepLifeSpan	Longevity of females sheep	6 to 11 years		[68]
lambAge	Age period for lamb	365 days		[68]
gestationPeriod	Period for foetus development	152 days		[68]
carryingCapacity	Environment’s maximum load	200 sheep per square kilometre	Calculated from sheep population in specific areas	
sheepAbortions	Foetus die per day due to RVF	90–100%		[69], [70]
lambMortality	Lamb die per day due to RVF	Less than 50%		[69], [70]
adultMortality	Adult sheep die per day due to RVF	20–30%		[69], [70]

### Parameter sensitivity and calibration

In order to understand the influence of many time based processes in this model, sensitivity analysis of model outputs due to variation in input parameters was assessed during simulation period. Stochastic random normal distribution was applied to quantify the sensitivity of the outputs [51]. Sensitivities were assessed on daily time-step spanning 100 steps of simulation. The influence of temperature, water level thresholds, infection period, incubation period and vector-host interactions on time-dependent sensitivities were quantified. Generated sensitivity data was then used to calibrate the models output to reflect the actual number that would have been trapped in the same period based on independent mosquito population dataset [52].

## Results

**Figure 2:**
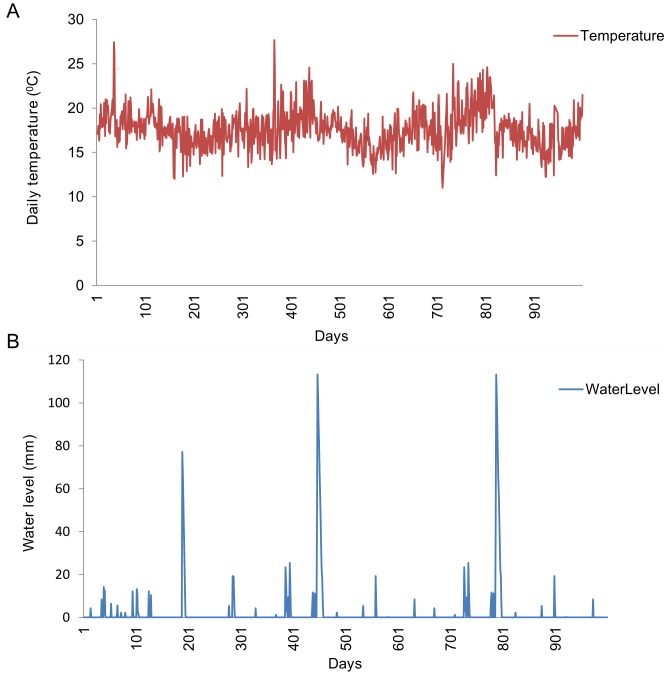
Daily weather data for Ngorongoro district from 1994–1999 only for the first 1000 days. (A) Daily temperature in °C. (B) Water level in breeding sites (millimetres) calculated from daily rainfall data.

During simulations, the following initial conditions were prescribed to run once; 50 adult Floodwater Aedine and Culicine mosquitoes that were allowed to lay eggs and initiated growth to larvae, pupae and adult mosquitoes under appropriate conditions. Similarly, initial population for host sheep was 50 lamb and 100 adults with the environmental carrying capacity of 200 sheep per square kilometer (Table 1). Mosquito population dynamics simulated for both floodwater Aedines and Culicine showed relationship with daily temperatures and rainfall fluctuate over a period from 1994 to January 1999 (Figure 2A) and rainfall data used to determine estimated amount of water in breeding sites for Aedine mosquitoes (Figure 2B).

Selected parameters were sensitive to substantial changes with vector-host population’s simulation time. Low temperature thresholds had a significant impact on larvae by delaying transfer of larvae to pupae. High temperature caused high mortality in larvae and reduces adult survival days. Water level thresholds that depended on daily rainfall influenced the emergence of floodwater Aedine mosquitoes and hatching of infectious eggs laid in the soil above water level in the breeding site. At low water level thresholds, population of floodwater Aedine mosquitoes varied similar with Culicine which did not depend on water level for mosquito emergence. In this light, water level threshold for emergence of infectious floodwater is adjusted to reflect the biological role of floodwater Aedine in RVF epidemics.

Mosquito attack rates for blood meal and infection introduction during vector-host interactions were sensitive to determine stage and phase flows. Vector-host interactions were influenced by infection period and RVFV incubation period in hosts. Infectious period in mosquito vectors and hosts influenced the pattern and peak size of the simulated epidemic, longer infectious period extended the duration of the epidemic. For mosquitoes, this duration was set to the lifespan of the mosquito in order to reflect the actual duration of RVF epidemic whereas in hosts ranged from three to six days. Longer disease incubation period within hosts showed a delayed increase in the number of infectious hosts and therefore a later peak in the epidemic than when the incubation period is assumed to be short. Knowing the infection and incubation period appeared to be important in predicting dynamics of a simulated epidemic.

**Figure 3:**
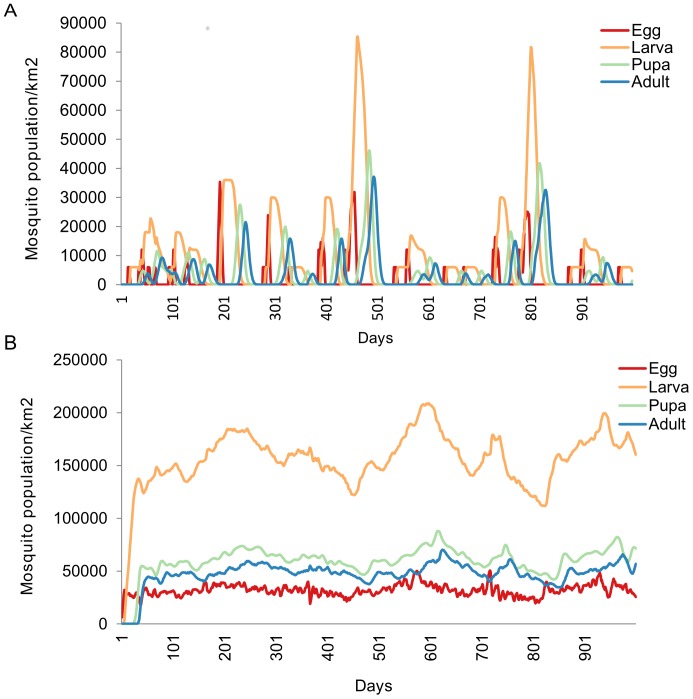
Simulated RVF vector population dynamics showing developmental stages from eggs, larvae, pupae and adults. (A) Floodwater Aedines depending on water level in breeding sites and host availability. (B) Culicines.

**Figure 4:**
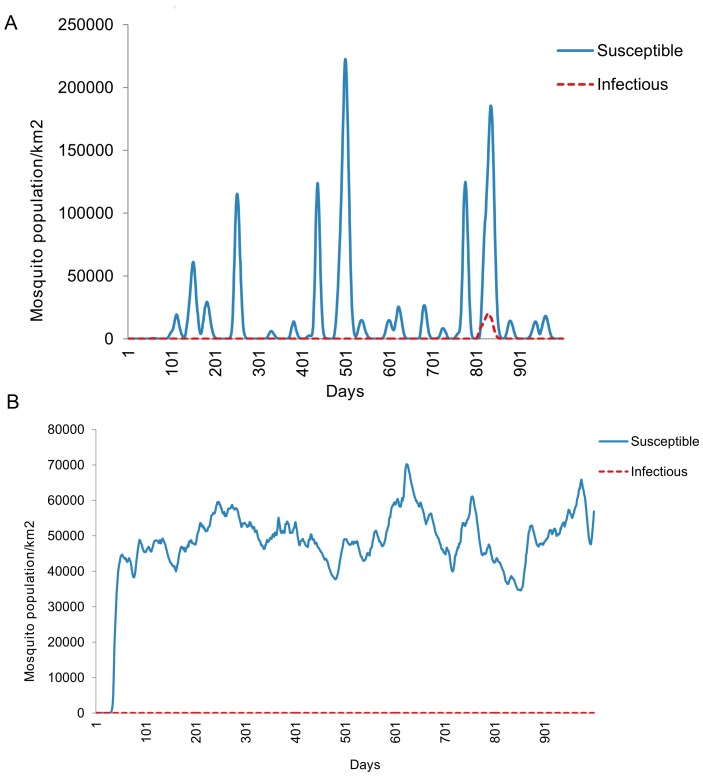
Simulated infection initiation before virus amplification. (A) Floodwater Aedines initiate infection. (B) Culicines without infection from infected sheep to prevent virus amplification.

Simulation results yielded equilibrium over time with a stable and consistent number of mosquitoes, regardless of the initial starting point of the adult population following model calibration. Floodwater Aedines and Culicine vector population continued to fluctuate with temperature and water level over the entire period while controlled by availability of host for blood feeding (Figure 3A, B). In order to initiate infection, emergence of infectious floodwater Aedines was set at different water level threshold (Figure 4A, B). Culicines only pick infection from infected sheep once in order to amplify disease epidemic (Figure 4B).

**Figure 5:**
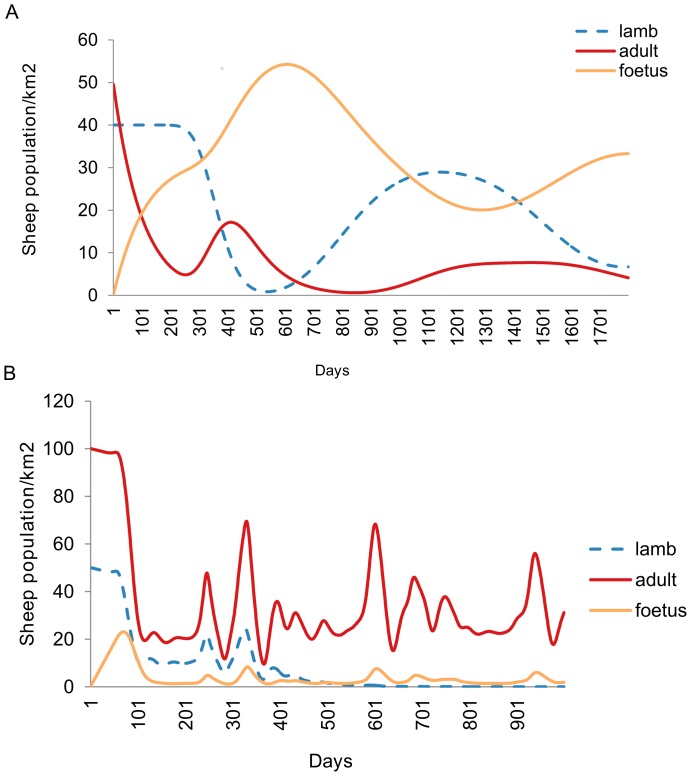
Sheep population dynamics controlled at the environmental carrying capacity of 200 sheep per square kilometre. (A) Growth stages without infection. (B Growth stages after introduction of controlled infection within Aedines mosquitoes only.

**Figure 6:**
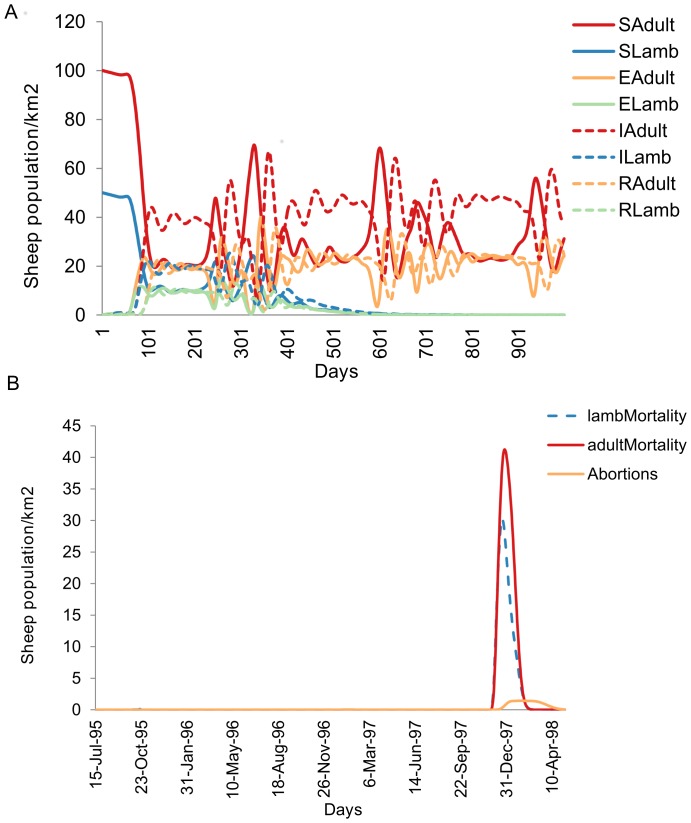
Simulated RVF epidemic. (A) Compartmental phases after allowing infection to flow from Aedines to Culicines for virus amplification, recovered hosts are not allowed to flow back into the susceptible hosts. (B) Calculated host mortality per developmental stages due to infection with RVF virus. **Abbreviations**: SAdult = Susceptible adults, SLamb = Susceptible lamb, EAdult = Exposed adult, ELamb = Exposed lamb, IAdult = Infectious adult, ILamb = Infectious lamb, RAdult = Recovered adult, RLamb = Recovered lamb.

Sheep population provided as lamb and adults were allowed to fluctuate over the whole simulation period. Sheep remained in the susceptible phase until had contact from infectious Floodwater mosquitoes (Figure 5A, B). Following infection introduction in the exposure phase, sheep were allowed to flow to infectious and recovered phases. Mortality due to disease was also calculated and simulated at a given time. Mortality provided varied with age group of sheep and sex to indicate high abortions in natural environment as indicator for RVF epidemic (Figure 6).

**Figure 7:**
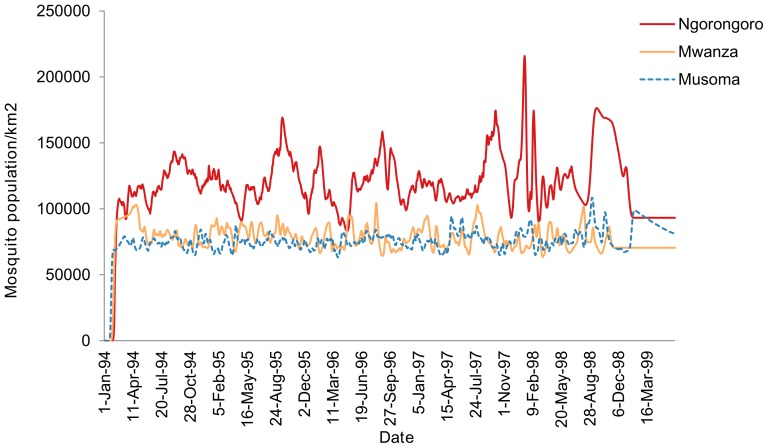
Vector population dynamics simulation results indicating unusual increase in mosquito population in late 1997 and early 1998. Similar period to when large-scale RVF outbreak occurred in Ngorongoro district. Disease outbreak did not occur in Mwanza and Musoma areas during the same period.

Controlled simulation of mosquito population dynamics without influence of host availability for a period from 1994 to 1999 showed sudden increase after about 1450 days of simulation, a period between December 1997 and January 1998, similar to the time in which Ngorongoro district experienced a RVF disease outbreak (Figure 7). However, this sudden increase in mosquito population was not observed in Mwanza region where RVF outbreak did not occurred in the same period (Figure 7). This unusual pattern in vector population increase could be associated with potential of RVFV outbreaks. The early stage of the simulated potential disease outbreak was characterized by an abnormal decline in vector population as a potential future epidemic indicator.

## Discussion

Our simulation modelling strategy was to produce useful tool for studying effects of daily rainfall and temperature on vector life stages in terms of stage-specific growth and death rates. Conditions that result in unusual abundance of vector mosquito species have been shown to have a positive association with RVFV epidemics [53]. The model that we developed provides understanding of the dynamics of RVFV vector population by implementing time dependent distribution delay and functional response modelling approaches for aggregated systems. These models have previously had broad applications in predicting life cycles of insects, animals, plants, trees, and capital goods in economics, but not in previously published RVF modelling papers [43], [46], [49]. Assessment of the value of the underlying biological processes allows us to examine potential variability in RVFV infections in animal and human populations given the vectors for both maintenance and amplification of the virus in the population.

Studying RVFV transmission dynamics poses a big challenge among scientists, as disease outbreaks are associated with abnormal changes in weather conditions which are essential components for prediction of disease epidemics. Choosing the right modelling procedure for this complex disease can be quite challenging. Simulation modelling of RVFV vector populations dynamic remains a useful tool in understanding these transmission dynamics. In our simulation model, we attempt to replicate the actual biological processes related to dynamics of the relevant disease vectors in a local endemic setting. This model takes advantage of previously developed mathematical equations for modelling disease vectors and hosts stages at different phases of infection but with careful selection of useful parameter in relation with the biology of RVFV [54]–[56].

The current procedures for simulations development are highly flexible to allow inclusion of factors that might accelerate the emergence and decline of Aedine population by not only considering availability of water in respective breeding sites. However, we carefully avoided including other factors such as landscape features [57] and soil types in relation with vectors distribution. Although Aedine populations may play important role of RVFV infection initialization, we limited our simulation procedure to generalized presentation of vectors for maintenance of the virus by Aedines and amplification during epidemics by Culicines. Despite this simplicity, our model produced reasonable vector population values and generated trajectories that were consistent with expectations for years from 1994 to 1999 based on only freely accessed weather data. This model provides flexibility for inclusion of more hosts and vectors interactions as would appear in disease epidemics setting.

While developing models for RVF disease prediction is highly useful, significant work is needed for further improvement in this modelling approach. We agree on simplification of some parameter estimation such as trans-ovarial transmission within Aedes mosquitoes due to the lack of more information. RVF virus vertical transmission in Aedes mosquitoes is still not known [31]. This simulation required inclusion of more relevant numerical information and use of the advanced calendar module in order to be able to clearly mark simulation dates linking biological processes with simulation output. Further model improvements should include need for separate models handling animal and human population dynamics and addition of spatial distribution of vectors and host in relation to disease distribution.

Simulation outputs from this study provide new insights for weather-driven RVFV epidemic modelling. This study shows that daily temperature and rainfall are key ecological factors to include in models that predict episodes related to RVFV outbreak [20]. Simulations provide an ideal approach for understanding the important parameters in virus transmission dynamics with important insights to be gained in prevention and control of such epidemics. This approach can be used as an alternative source for generation of RVFV epidemics data in different scenario for use in advanced computational analyses and can be modified for use to other diseases. Final version of this simulation model is available for download as a Universal Simulator plug-in in both the end user version and source code from http://www.ecolmod.org/download.html.

## Supporting Information

Text S1 File containing instructions to guide installation and use of therein attached RVF plug-in source codes.
(ZIP)
