# Supplementary material for: Simulation Modelling of Population Dynamics of Mosquito Vectors for Rift Valley Fever Virus in a Disease Epidemic Setting
Source: PLoS One. 2014 Sep 26;9(9):e108430. doi: 10.1371/journal.pone.0108430 (PMC4178157; doi:10.1371/journal.pone.0108430)
Supplement: Text S1 — File containing instructions to guide installation and use of therein attached RVF plug-in source codes. (ZIP) [file pone.0108430.s001.zip › RVFPlugin_sourceCodes/instructions.docx]

**Instructions**

This is a readme file with instructions to guide installation and use of RVF plug-in source codes. This zip file contains source codes for RVF plug-in, xml file and daily rainfall and temperature data for Musoma, Mwanza and Ngorongoro-Narok ecosystem.

Requirements

- End-user version together with its developers version of the Universal Simulator (<http://www.ecolmod.org/download.html>)
- Qt development environment (<http://qt-project.org/downloads>) and
- Text editor such as Komodo edit (<http://www.activestate.com/komodo-edit/downloads>) or notepad++ (<http://notepad-plus-plus.org/download/v6.5.5.html>)

Download the Universal Simulator explained book (<http://www.ecolmod.org/download.html>) and follow instructions on how to install the developer’s version of the Universal Simulator.

After successful installation of all required software:

- Copy the RVF plug-in source codes provided here and replace it with the older versions in the developers version of the Universal Simulator
- Copy the xml file with its input data text files then add to the recipe accordingly

You can now run your simulations!

To cite the source of the UniSim software write: 'Holst N (2012). Universal Simulator 1.39. Aarhus University, Aarhus, Denmark, www.ecolmod.org'. State the version number and year corresponding to your version. You can find it under the Help|About menu of UniSim.

The RVF plug-in within Universal Simulator is maintained by Clement Mweya ([mweyaclement@gmail.com](mailto:mweyaclement@gmail.com) or [cmweya@nimr.or,tz](mailto:cmweya@nimr.or,tz)), National Institute for Medical Research, Tukuyu Research Centre, P.O. Box 538, Tukuyu, Tanzania

**Note**: Our model is a plug-in within the universal simulator. It was developed using open source software, universal simulator (UniSim) and Qt Creator integrated development environment therefore making it open source too. Data for this simulation modelling procedure can freely be accessed from <http://www.ecolmod.org/download.html>.
